# A Bibliometric Review of Publications on Oxidative Stress and Chemobrain: 1990–2019

**DOI:** 10.3390/antiox9050439

**Published:** 2020-05-18

**Authors:** Taylor McElroy, Antiño R. Allen

**Affiliations:** 1 Division of Radiation Health, University of Arkansas for Medical Sciences, Little Rock, AR 72205, USA; TMMCELROY@uams.edu; 2 Department of Pharmaceutical Sciences, University of Arkansas for Medical Sciences, Little Rock, AR 72205, USA; 3 Department of Neurobiology & Developmental Sciences, University of Arkansas for Medical Sciences, Little Rock, AR 72205, USA

**Keywords:** oxidative stress, chemotherapy, antioxidants, chemobrain, cognitive dysfunction

## Abstract

Oxidative stress is considered one of the possible mechanisms behind chemobrain or the cognitive dysfunction persistent after chemotherapy treatment. Breast cancer patients have reported chemobrain symptoms since the 1990s. In this present bibliometric review, we employed the VOSviewer tool to describe the existing landscape on literature concerning oxidative stress, breast cancer chemotherapies, and chemobrain. As of 2019, 8799 papers were listed in the Web of Science database, with more than 900 papers published each year. As expected, terms relating to oxidative stress, mitochondria, breast cancer, and antioxidants have occurred very often in the literature throughout the years. In recent years, there has been an increase in the occurrence of terms related to nanomedicine. Only within the last decade do the keywords ‘brain’, ‘blood-brain barrier’, and ‘central nervous system’ appear, reflecting an increased interest in chemobrain. China has become the most prolific producer of oxidative stress and chemotherapy related papers in the last decade followed by the USA and India. In conclusion, the subject of oxidative stress as a mechanism behind chemotherapies’ toxicities is an active area of research.

## 1. Introduction

Cancer has been a popular topic of bibliometric analyses; these investigations have examined multiple types of cancer, technological impacts, translational medicine, and immunotherapy [1,2,3,4,5,6,7]. Whether broad or specific, the goals are to describe the landscape of cancer research. Breast cancer, its chemotherapies, and oxidative stress are the focus of this bibliometric review, and more specifically, exploring how it fits into the phenomenon of chemobrain. Bibliometric reviews on breast cancer have analyzed both short- and long-term research outputs [8,9]. Reviews have covered research outputs for countries, organizations, such as the National Breast Cancer Foundation, and global trends [10,11,12,13,14]. Topics have considered therapeutic options including gene therapy, anti-VEGF (vascular endothelial growth factor) therapy or monoclonal antibodies to inhibit tyrosine kinases stimulated by VEGF, nanomedicine, diet, and natural products [15,16,17,18,19]. For the sake of this review, we chose to do a long-term (30 years) study to investigate trends over time.

In the early 2000s, breast cancer patients’ self-reported difficulties brought the cognitive side effects of chemotherapy to oncology’s attention [20]. Breast cancer is the most commonly diagnosed cancer in women globally and the leading cause of cancer death [21]. In 2018, an estimated 2 million breast cancer cases were diagnosed worldwide. The incidence is expected to continue to rapidly rise in part due to aging, a growing population, along with the many risk factors [21]. In 2019, the United State estimated 3.8 million women living with a history of invasive breast cancer [22]. While the number of cases increases, so does the number of breast cancer survivors. Early detection and advances in treatment contribute to this. For example, the United States’ 5-year survival rate is nearly 100% for patients diagnosed with Stage I cancer [22]. Adjuvant chemotherapy is used in both early and late-stage breast cancer to lessen the chance of the cancer returning; however, there are significant side effects both short-term and long-term [23]. 

Combination chemotherapy started in the 1970s to treat early stage breast cancer that included 12 cycles of cyclophosphamide, methotrexate, and 5-fluorouracil (CMF). The 1980s saw the addition of anthracyclines (Adriamycin, epirubicin) to the cyclophosphamide and 5-fluorouracil regimen [23]. In the United States, four cycles of doxorubicin and cyclophosphamide (AC) became common breast cancer treatment after showing similar results to six cycles of CMF [24]. By the 1990s–2000s, four cycles of the taxane paclitaxel followed four cycles of AC to create the AC-T chemotherapy regimen [24,25]. Short-term side effects of AC-T include nausea, vomiting, mucositis, fatigue, alopecia, neutropenia, neuropathy, and myalgia [26,27,28,29]. These short-term side effects typically resolve with the completion of chemotherapy. Long-term side effects include premature menopause or amenorrhea, increased risk for osteoporosis, and cardiac effects [22,29]. Paclitaxel has been associated with neuropathy which can persist after treatment [30]. Chemotherapy-associated leukemia is rare, but has been seen in patients treated with AC-T [29]. Chemotherapy-induced cognitive impairment, colloquially known as chemobrain, is the long-term side effect in focus of this review. Despite knowing about this phenomenon for at least 20 years, there still is a lack of knowledge behind the mechanism. This also results in zero treatments for chemobrain. Considering oxidative stress as mechanism behind chemobrain, future avenues into antioxidant research as therapeutics is vital.

Investigators now tend to address chemobrain as a consequence of chemotherapy, but there is still research needing to be done to address cognitive decline because of cancer itself. A perturbed balance between reactive oxygen species (ROS) and antioxidants is known as oxidative stress. Several chemotherapy agents have been shown to cause oxidative stress both peripherally and in the central nervous system. Doxorubicin and cyclophosphamide, a common regimen for breast cancer treatment, individually have increased oxidative stress in the brains of rodent models [31,32,33,34,35]. Oxidative stress as a mechanism behind chemobrain has been proposed acting mainly through mitochondrial dysfunction resulting in reduced cellular energy [36].

Although oxidative stress surfaced in previous reviews concerning oncological research and the neurotoxicity of nanoparticles, there has yet to be a bibliometric analysis reviewing the research output on publications concerning oxidative stress as a mechanism behind chemotherapy-induced toxicity to the brain (i.e., chemobrain). Bibliometrics are statistical methods used to analyze publications and have been used to measure the output of individuals, institutions, and countries [37]. The impact of research can be explored, as well as the trends over time within the disciplines. Contributions of topics in a particular field of study can be investigated using keyword and citation analysis [38,39]. We evaluated the existing literature on oxidative stress and chemobrain by performing a bibliometric analysis focused on: (1) the most common terms mentioned in chemobrain and oxidative stress-related papers, and (2) research volume output by authors, institutions, country, and journals over three decades (the 1990s, 2000s, and 2010s), including an assessment of impact.

## 2. Materials and Methods 

### 2.1. Data Source and Search Strategy

This bibliometric study analyzed research articles related to oxidative stress, oxidative species, cancer, chemotherapy, and chemobrain published from 1 January 1990 through 31 December 2019. The study period was subdivided into three decades (1990s, 2000s, and 2010s) to analyze temporal trends. The Web of Science online database (Clarivate Analytics, Philadelphia, PA, USA) was accessed January 2020. Two sets of separate topic searches were performed under the advanced search option. “ts” denotes a topic search which returns results pertaining to the title, abstract, and keywords of publications. The search terms for each set are as follows:

Set 1: ts = (“reactive oxygen species” or ROS or superoxide or “hydrogen peroxide” or H_2_O_2_ or “hydroxyl radical”)

Set 2: ts = (chemobrain or “chemo brain” or “chemo-brain” or “breast cancer” or doxorubicin or cyclophosphamide or paclitaxel)

Both sets included the following Indexes: Science Citation Index Expanded, Social Sciences Citation Index Timespan = 1990–2019. The two sets were combined with the ‘AND’ Boolean operator. Excluded from the results were proceedings papers, book chapters, and retracted publications. Data was extracted and downloaded as full records and cited references in tab-delimited text files. The type of data included publication year, authorship, publication title, abstract, author keywords, citation count, journal title, institution, and country.

### 2.2. Data Analyses and Presentation

For bibliometric analysis, data were imported into VOSviewer version 1.6.14 (Centre for Science and Technology Studies, Leiden University, Leiden, The Netherlands) [40]. Term maps were created using the following option/commands: “Create a map based on bibliographic data”, “read data from bibliographic database files”, “type of analysis: co-occurrence”, “unit of analysis: all keywords”, and “counting method: full counting”. Full counting means that a term having multiple occurrences in a single publication is counted as one. A thesaurus file was created using the top 5000 common words from the Corpus of Contemporary American English to exclude from the analysis as previously described [41,42]. The thesaurus was appended with additional general terms to exclude terms such as ‘method’ and ‘result’. Preliminary term mapping produced a list that included hyphenated terms.

The thesaurus was further appended so the software would recognize terms such as ‘lipid-peroxidation’ and ‘lipid peroxidation’ as the same term. The thesaurus also required manual curation to ensure that terms relating to brain and cognition were included in the analysis. See Table S1 for the final thesaurus used. The software performs keyword analysis by identifying keywords in the titles and abstracts of publications and relating them to documents in which they occur together (co-occurrence analysis). The co-occurrence frequencies measure the relatedness of terms. Thresholds are placed to sort through all the keywords. The default threshold is at 5 where the minimum number of occurrences of a keyword appears in a single document is 5 times. The minimum occurrence for keywords were the following per period: 1990s—10×; 2000s—12×, 2010s—15×. The occurrence was increased due to the increase in the number publications while including terms related to the brain. The parameter of ‘averaged citations’ was used for visualization purposes indicating the average number of citations received by the documents in which a term occurs. VOSviewer software constructed a 2D term map based on the co-occurrence frequencies.

For 1990–2019, countries’ publication and citation data were extracted on 5 February 2020. For citation analysis, the 159,235 citations that occurred without self-citation were used. A map illustrating the countries data for the related papers was created using the 3D Map feature in Excel 2016 (Microsoft, Redmond, WA, USA).

## 3. Results

### 3.1. Output of Research on Oxidative Stress, Chemotherapy, Cancer, and Chemobrain in the Form of Publications Has Been Increasing from 1990 to 2019

**Figure 1:**
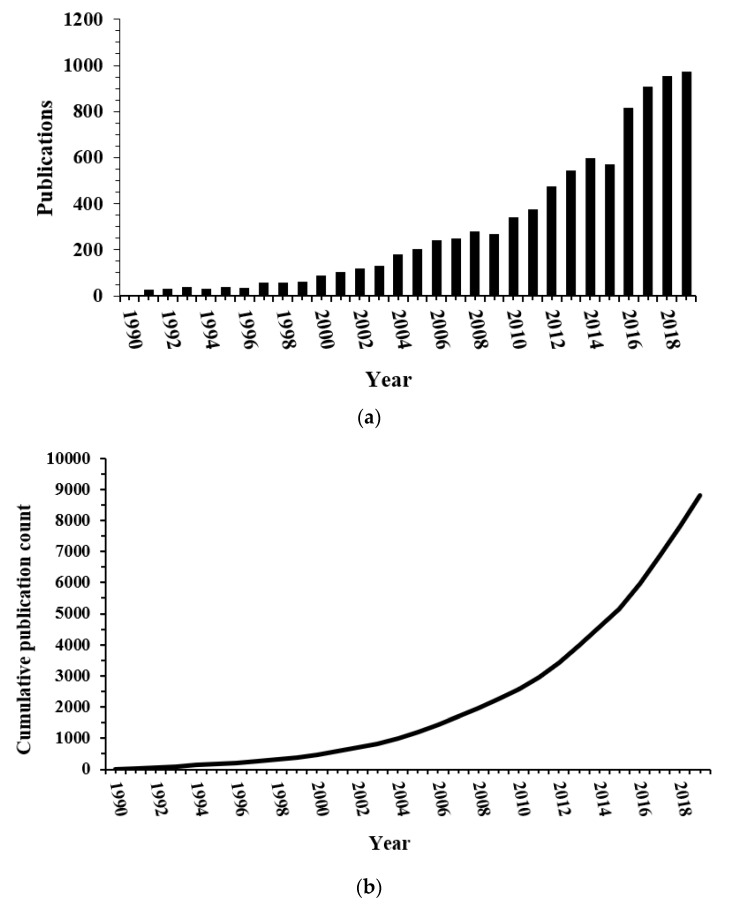
(**a**) Number of publications about oxidative stress and chemobrain per year. (**b**) Cumulative number of publications about oxidative stress and chemobrain per year.

The Web of Science search generated 8799 publications. Separated into time periods, the 1990s had 380 publications, the 2000s had 1859 publications, and the 2010s had 6560 publications. There were only four publications in 1990. In 1994, there were over 100 publications. Publications exceeded 1000 cumulatively in 2005. Since 2017, there have been between 900 and 1000 publications per year (Figure 1a). There have yet to be more than 1000 publications in any year; however, there was exponential growth of publications across the 30-year period, especially within the last decade. The cumulative number of publications indicates increasing activity in research into the connection between cancer, chemotherapy, and oxidative stress (Figure 1b).

### 3.2. Evolution Over Time of Bibliographic Terms Occurring in Related Publications

**Figure 2:**
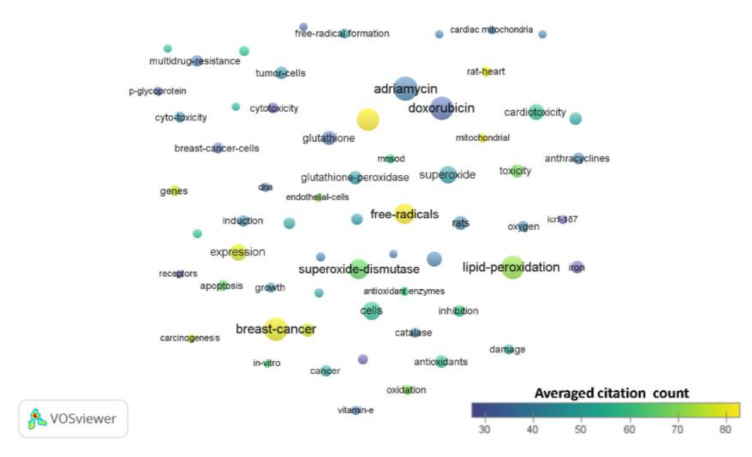
Term map for years 1990–1999. Term map showing the visualization of 63 terms that occurred at least 10 times in documents between 1990 and 1999. Each circle represents a term. The size of the circle is proportional to the occurrences of the term (the bigger the circle, the higher the number of occurrences). Please refer to the color scale indicating averaged citation count. The proximity of circles indicates the frequency of co-occurrence between the two respective terms (the closer the proximity, the higher the frequency). Table S2 contains all the terms visualized with their respective occurrence frequencies and averaged citations.

**Figure 3:**
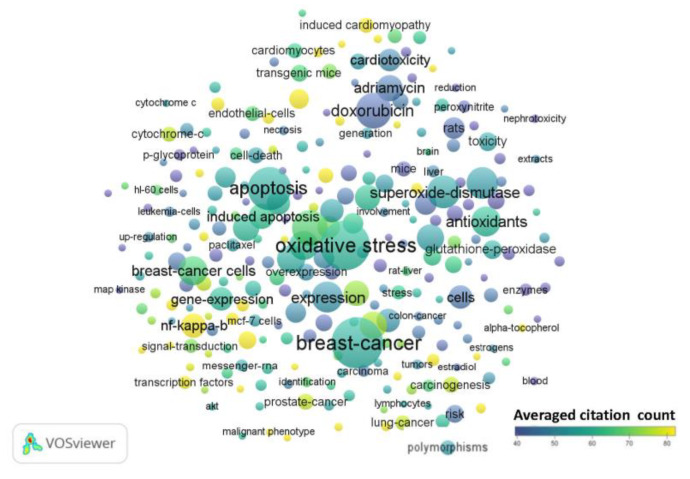
Term map for years 2000–2009. Term map showing the visualization of 262 terms that occurred at least 15 times in publications between 2000 and 2009. Each circle represents a term. The size of the circle is proportional to the occurrences of the term (the bigger the circle, the higher the number of occurrences). Please refer to the color scale indicating averaged citation count. The proximity of circles indicates the frequency of co-occurrence between the two respective terms (the closer the proximity, the higher the frequency). Table S3 contains all the terms visualized with their respective occurrence frequencies and averaged citations.

**Figure 4:**
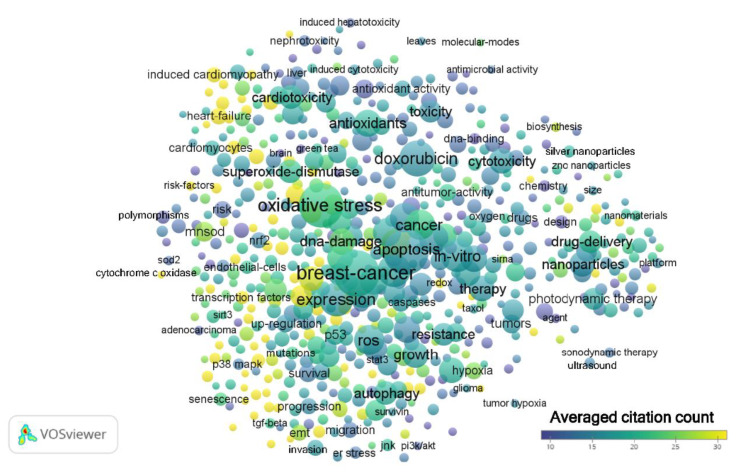
Term map for years 2010–2019. Term map showing the visualization of 605 terms that occurred at least 15 in publications between 2010 and 2019. Each circle represents a term. The size of the circle is proportional to the occurrences of the term (the bigger the circle, the higher the number of occurrences). Please refer to the color scale indicating averaged citation count. The proximity of circles indicates the frequency of co-occurrence between the two respective terms (the closer the proximity, the higher the frequency). Table S4 contains all the terms visualized with their respective occurrence frequencies and averaged citations.

After analysis of each period using VOSviewer, the generated term maps for each time period are shown in Figure 2, Figure 3 and Figure 4. From the 1990s results, there were 63 terms with 3 clusters, 1132 links or connections, and 3391 total link strengths (Figure 2, Table S2). The most frequent terms were ‘breast-cancer’, ‘adriamycin’, ‘doxorubicin’, ‘lipid-peroxidation’, ‘superoxide-dismutase’, ‘hydrogen-peroxide’, and ‘free radicals’. The terms brain, ‘blood–brain barrier’ and ‘CNS’ do not appear for this period. The term ‘mitochondrial’ presents in cluster 2 with 33 links, total link strength of 74, and occurs 12 times (Table S2). From the 2000s results, there were 262 terms with 5 clusters, 12,465 links or connections, and 39,312 total link strengths (Figure 3, Table S3). The most frequent terms were ‘oxidative stress’, ‘breast cancer’, ‘antioxidants’, ‘apoptosis’, ‘hydrogen-peroxide’, ‘doxorubicin’, and ‘superoxide dismutase’. The terms ‘blood–brain barrier’ and ‘CNS’ do not appear for this period. The term ‘brain’ presents in cluster 2 with 50 links, total link strength of 95, and occurs 12 times (Table S3). The term ‘mitochondrial’ presents as a central node in cluster 1 with 178 links, total link strength of 816, and occurs 111 times. From the 2010s results, there were 605 terms with 7 clusters, 48,211 links or connections, and 187,829 total link strengths (Figure 4, Table S4). The most frequent terms were ‘breast-cancer’, ‘apoptosis’, ‘oxidative stress’, ‘doxorubicin’, ‘expression’, and ‘in vitro’. The term ‘brain’ presents in cluster 2 along with the terms ‘blood-brain barrier’, ‘dysfunction’, ‘CNS’, and ‘mitochondrial dysfunction’ (Table S4). Interestingly, the term ‘mitochondrial’ is in cluster 1 with no links and occurs 434 times (Table S4).

Common terms appear throughout all three decades as indicated by relative node size indicating the number of times the term occurs. These include ‘doxorubicin’, ‘breast cancer’, and ‘lipid peroxidation’. Oxidative stress, of course, is the main theme throughout because of the nature of the topic search. Key terms that confirm this include ‘oxidative stress’ itself, ‘free radicals’, ‘hydrogen peroxide’, and ‘superoxide dismutase’, which are all players in oxidative stress. Terms related to mice and rats appear throughout the years (Tables S2–S4), denoting the importance and frequent use of these animal models in chemotherapy and cancer related studies. The term ‘cells’ also appears, denoting in vitro studies involving cancer cells. The organ that appears the most in the term maps throughout the years is the heart. Other toxicities that occur in the term maps include hepatotoxicity and nephrotoxicity. A new cluster emerges in the 2010s regarding chemotherapies, delivery systems, nanoparticles, and photodynamic therapy that endorses these emerging research interest areas in cancer treatment.

### 3.3. Evolution of Oxidative Stress and Chemobrain-Related Research in Terms of Countries of Origin

**Figure 5:**
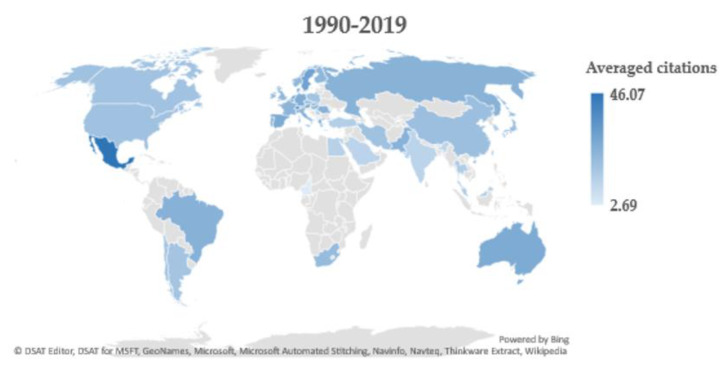
World map depicting the averaged citations per oxidative stress and chemotherapy-related papers published 1990–2019. Papers may have authors from more than one country denoting international collaboration. Please refer to the color scale for averaged citations. Publications from England, Scotland, Wales, and Northern Ireland were combined into ‘United Kingdom’ for visualization purposes. Countries with at least 20 publications are depicted. See Table S5 for full details.

Initially between 1990 and 1999, papers about oxidative stress, cancer, and chemotherapy originated mainly in the USA (~50%) followed by Japan (~10%) (Table 1). Canada, France, and England also provided a considerable amount of research. During this time the USA produced the most citing articles (6241) (Table 2). China, Japan, Germany, and Italy followed all relatively close in rank (Table 2). Between 2000 and 2009, the USA and Japan were still the top two ranking countries with USA at approximately 40% and Japan at approximately 8%. China was a close third with 7% of the total papers, followed by India and South Korea (Table 3). The USA continued to rank first in production of 6241 citing articles (Table 4). China ranked second with 13,411 citing articles. Italy, Japan, and India finished up the top five all close in total number of citing articles (Table 4). From 2010 to 2019, China led as the number one country, producing approximately 30% of the total papers (Table 5). The USA produced approximately 23%, India approximately 10%, South Korea approximately 7%, and Italy approximately 4%. Thus, research into oxidative stress and cancer chemotherapy is worldwide, and it is noteworthy that a large volume now comes from China, followed by the USA. Similarly, China lead in the production of the most citing articles (1920) with the USA ranking second with 1476 articles (Table 6). India and Italy remain in the top five countries producing citing articles with the addition of South Korea placing fourth (Table 6). As an index of relative impact, Figure 5 depicts averaged citations per publication in each country with at least 20 published papers for the whole period of analysis (1990–2019). The leading countries in this aspect are Mexico, Russia, Brazil, Australia, Canada, USA, Egypt, Saudi Arabia, South Africa, and Pakistan. Table S5 includes the average citation data per document for each country next to the total number of publications.

### 3.4. Profile of the Top 5 Productive and Most Cited Authors 1990–2019

For the 1990s, B.B. Hasinoff ranked first with 12 publications followed by L.W. Oberley with 10 publications (Table 7). Ferrans, Gutierrez, and Herman all ranked third with 6 publications each (Table 7). Oberley and Hasinoff were the most cited authors (Table 8). In the 2000s, Oberley also ranked second right behind C.B. Ambrose with 18 publications (Table 9). D.R. Spitz ranked third with 16 publications. B. Kalyanaraman and J. Joseph placed fourth and fifth (Table 9). The most productive authors did not show up in the top five most cited authors (Table 10). The 2010s, see Y. Zhang rank first with 76 publications (Table 11). Y. Wang and Y. Liu rank closely at second and third followed by Y. Li and J. Li (Table 11). The four most productive authors were the four most cited authors, keeping the same ranking (Table 12).

### 3.5. Profile of the Top 5 Productive and Most Cited Journals 1990–2019

In the 1990s, Biochemical Pharmacology produced the most publications (5%) on the related topics followed closely by Cancer Research and Free Radical Biology and Medicine (Table 13). Archives of Biochemistry and Biophysics and Chemical Research in Toxicology tied for fourth place with 10 publications each around 2.6% of the total publications (Table 13). Free Radical Biology and Medicine ranked second in journals with citing articles (Table 14). Cancer Research ranks third with 263 citing articles (Table 14). In the 2000s, Cancer Research placed first with 78 publications (4.2%) followed closely by Journal of Biological Chemistry (Table 15). Free Radical Biology and Medicine ranked third again with 54 publications. International Journal of Cancer and Oncogene ranked fourth and fifth respectively (Table 15). As for the journals with citing articles, PLoS One ranks first with 1664 articles (Table 16). Free Radical Biology and Medicine, Journal of Biological Chemistry, and Cancer Research rank second, third, and fourth respectively (Table 16). The 2010s saw PLoS One rank first in publications with 198 or 3% of all publications (Table 17). Oncotarget and Scientific Reports ranked second and third respectively (Table 17). Free Radical Biology and Medicine went down to fourth in rank with ~1.6% of total publications followed by International Journal of Molecular Sciences (Table 17). The top three most productive journals were also the top three journals with citing articles (Table 18). International Journal of Molecular Sciences ranks fourth with 1181 citing articles (Table 18).

### 3.6. Profile of the Top 5 Productive and Most Cited Organizations 1990–2019

For the 1990s, the five most productive organization in terms of research output in the form of publications were all from North America (Table 19). The National Institutes of Health (NIH) ranked first and the NIH’s National Cancer Institute (NCI) as third (Table 19). The search in Web of Science was done using their organization-enhanced field. This used a unified list of preferred names that included many variants of an institution’s name. For example, the NCI is considered a part of the NIH; however, as a single institution the NCI produced a significant amount of publications and is included as a separate ranking. The University of Manitoba in Canada also ranked third with 14 publications (Table 19). Then, University of Texas and Minnesota Systems were ranked second and fourth respectively (Table 19). The “Systems” of institutions include the unified list of preferred names. For example, the University of Texas system umbrellas both academic and health institutions. Thus, University of Texas MD Anderson Cancer Center and the University of Texas at Dallas would fall under the University of Texas system. It is a similar case with other university systems.

The University of Texas system had the most citing articles (461) while the NIH and University of California system ranked close behind (Table 20). Moving into the 2000s, the NIH still ranked first with 73 publications but overall contributed less percentage wise than the previous decade (Table 21). This is due to the fact the number of publications increased from 380 in the 1990s to 1859 in the 2000s. The NCI moved to second in rank with 51 publications or 2.7% of the total publications. The University of Texas System moved to third place. The University of California System and University of Iowa ranked closely at fourth and fifth (Table 21). The same five institutions with the most citing articles in the 1990s were also in the top five for the 2000s although in different rankings (Table 22). It was not until the 2010s that other global organizations were ranking at the top five. The Chinese Academy of Sciences ranked first with 159 publications followed closely by the University of Texas System with 142 publications (Table 23). The Council of Scientific Industrial Research (CSIR) in India ranked third, the Shanghai Jiao Tong University fourth and the University of Texas MD Anderson Cancer Center fifth (Table 23). The Chinese Academy of Sciences also ranked first in citing articles with 2332 articles (Table 24). We still see the University of Texas System, Harvard, and the University of California System ranked second through fourth (Table 21). Shanghai Jiao Tong University ranks fifth with 1058 citing articles (Table 24).

## 4. Discussion

### 4.1. Trends in Oxidative Stress and Chemotherapy Research

This bibliometric review on oxidative stress and chemotherapy papers, particularly in regard to chemobrain to date have revealed intriguing and thought-provoking facts. First, the field is continually expanding seeing close to 1000 new publications per year (Figure 1). Whether this number will continue to increase, stay steady, or decline is yet to be seen; however, the increasing trend looks promising. The types of research areas are also growing as we see the addition of material sciences and biotechnology as reflected in the new cluster in the 2010s featuring nanoparticles (Figure 4). Basic science areas such as biochemistry/molecular biology, cell biology, pharmacology, and toxicology still play a major part of research alongside clinical areas (oncology and neurology). Terms related to model systems such as ‘cells’, ‘yeast’, ‘mice’, and ‘rats’ were present throughout supporting basic research. The gradual inclusion of more clinical terms such as ‘Parkinson’s disease’ in the 2000s and ‘Alzheimer’s disease’ in the 2010s reflect interest in the commonalities between neurological diseases and chemobrain. It is striking that terms relating to the brain did not show up until the 2010s given the cognitive side effects of breast cancer chemotherapy were known since the early-mid 2000s [20]. 

The appearance of ‘senescence’, ‘cellular senescence’, and ‘premature senescence’, in the 2010s hints at possible mechanisms behind chemobrain (Table S4). Cellular senescence, a hallmark of aging, is a natural biological process in response to a variety of stresses [46]. Senescence is marked by permanent growth arrest, distinct morphological features, and secretory phenotype. The process promotes tissue remodeling by getting rid of unwanted cells [47], but also serves as a barrier to malignant tumorigenesis [48]. As anticancer treatment, pro-senescent therapies could be beneficial. Chemotherapy, in addition to inducing apoptosis in cancer cells, has been shown to force cancer cells into senescence [46]. Natural compounds that act as antioxidants can also induce senescence in cancer cells [49]. Vitamin E analogues are thought to be among those which may induce senescence [49]. Indeed, δ- and γ-tocotrienols were reported to reduce cell viability and induce expression of senescent-like growth arrest markers in breast cancer cell lines [50]. While having an anticancer effect on tumor cells, such natural compounds show a protective effect on normal, nontumor cells [49]; that would be an ideal scenario. Hence, using antioxidants as senolytics, or senescent-inducing agents, as adjuvant therapy is attractive. Most of these cytoprotective properties are thought to be mediated by Nrf2 (nuclear factor erythroid-derived 2 related factor 2) [46]. As an oxidative stress system, the Nrf2-Keap1 (Kelch-like ECH-associated protein 1) while protecting normal cells presents a paradox regarding tumor cells. Cancers with high levels of Nrf2 are associated with poor prognosis and chemoresistance; however, activating Nrf2 earlier in tumorigenesis is considered protective while detrimental in later stages [51]. Thus, it is fascinating how Nrf2 activating compounds can induce senescence in cancer cells highlighting the need for more research in this area. The effects of senolytics on cognition and chemobrain would also be most welcome.

Besides the term ‘antioxidants’, other antioxidants do show up on the term maps with ‘vitamin E’ being constant throughout the whole period. All forms of vitamin E are potent antioxidants by scavenging lipid peroxyl radicals [52]. As previously mentioned, vitamin E analogues in both mechanistic and preclinical animal studies have been promising to preventing cancer progression in various types of cancer [53]. In addition, γ-tocotrienol has been shown to promote the anticancer effects of chemotherapy drugs such as DOX and paclitaxel via the downregulation of NF-kappaB dependent genes [54]. This suggests that vitamin E analogues may be useful as combination or adjuvant therapy to increase the effectiveness of chemotherapy drugs. ‘Alpha tocopherol’ shows up in the 2000s and 2010s term maps. This isoform of vitamin E is the predominant form found in tissues; however, animal and humans studies looking at its cancer preventive properties have been discouraging [52]. Beta-carotene also shows up in the 2000s term map. Although acting as an antioxidant and vitamin A precursor, supplementation with beta-carotene has shown to be harmful to at risk populations such smokers in developing lung cancer [55,56]. Indeed, studies on randomized controlled trials involving beta-carotene as a cancer-preventative do not recommend the supplementation [56]. ‘Flavonoids’, or naturally occurring polyphenols in plants, also show up in the 2000s and in the 2010s. ‘Polyphenols’ as term shows up as well in the 2010s along with ‘genestein’ an isoflavone and ‘quercetin’ a flavonol. Although there has been promising evidence that increasing dietary intake of flavonoids reduces the risk of certain cancers [57], there are challenges in the isolation, purification, and pharmokinetic properties which have limited its development as a clinical drug [58]. The last antioxidant supplement to show up in the 2000s term map was ‘zinc’ which also appeared in the 2010s. Zinc supplementation is associated with decreased oxidative stress; however, more preclinical and clinical studies are needed with zinc supplementation by itself in order to characterize its effectiveness as a chemo preventative agent [59]. Other natural products such as ‘resveratrol’, ‘curcumin’, and ‘berberine’ show up in 2010s term maps all of which are being investigated for their chemo preventative properties [60]. Resveratrol and DOX has been shown to induce premature senescence in human primary dermal fibroblasts. Interestingly, resveratrol has been shown to suppression DOX-induced cardiotoxicity [61]. The phytochemical *sulfurophane* also appeared in the 2010s. The Nrf2-activating agent was also shown to protect against DOX-induced cardiotoxicity [62]. ‘N-acetylcysteine’ (NAC) is another antioxidant to show up in the 2010s. As a precursor to the antioxidant glutathione, NAC has been investigated in treatment of vascular and nonvascular neurological disorders [63]. ‘Selenium’ shows up in the 2010s indicating its possible role in chemoprevention; however, studies of randomized controlled trials do not show evidence to support supplementation [64]. Ascorbic acid and vitamin C also showed up in the 2010s. Ascorbic acid has been shown to mitigate D-galactose induced brain aging in the hippocampus of mice which in part is thought to be due to its antioxidant effects [65]. The appearance of multiple antioxidants with regard to their chemo preventative properties and protective effects against aging suggest these compounds may be of use in the treatment of chemobrain.

The heart showed up consistently which is unsurprising, because doxorubicin (particularly along with cyclophosphamide) has cardiotoxic effects in anticancer therapy which have been acknowledged for almost 30 years. DOX and anthracyclines are among the drugs whose cardiotoxicity is most widely described [66]. Depending on the dose, pharmacokinetics, and type of anthracycline used, myocardial cell loss and/or functional impairment may occur [67,68]. Oxidative stress is thought to be primarily responsible for DOX cardiotoxicity [69]. Myocardial tissues lack sufficient antioxidant mechanisms however; it produces a high amount of basal pro-oxidants, which makes it susceptible to oxidative stress damage [70]. In addition, cardiomyocytes have a lower ability to regenerate [71] thus, they are more susceptible to the long-term adverse effects of DOX. 

Nanotechnology offers a variety of advantages for cancer therapy overcoming the problems of existing chemotherapeutic agents. Chemotherapeutic agents are limited by their narrow therapeutic window and high risk for toxicity [72]. Nanoparticles (NPs,) ranging in sizes from 10 to 1000 nm, have improved the delivery of many drug molecules including chemotherapeutic agents [73]. In addition, NPs have large functional surfaces which are able to bind, absorb and carry drugs and proteins [74]. Furthermore, traditional cancer chemotherapy drugs are non-selective; they migrate to almost all parts of the body via the bloodstream [75]. NPs can accumulate in the breast cancer microenvironment because of their improper structure or due to focusing antibodies to specific molecular targets located on tumor membranes [76]. Thus, NPs represent a technology that can enhance therapeutic efficiency in cancerous cells while sparing normal ones.

### 4.2. A Changing Global Landscape of Oxidative Stress and Chemotherapy Research

There was a major shift in output in terms of volume from USA and Japan to China. China’s considerable increase in research output shifted the percentage of the total global output. This does not mean that the USA and Japan produced less research related to oxidative stress and chemotherapy when considering absolute values; rather, it is their relative contribution to the percentage of the total global output that decreased. There were also substantial contributions to research output by other European and Asian countries.

### 4.3. Author Productivity and Citation Impact

The top authors results were interesting in that they coincided along with the countries’ data and identified individuals that came from the same lab or collaborating labs. L.W. Oberley appeared in the top charts from 1990–2009. D.R. Spitz was from the same lab/institution and was alongside Oberley in the 2000s top productive authors chart. B. Kalyanaraman and J. Joseph ranking fourth and fifth were both from the Medical College of Wisconsin. There was a shift where four out of five of the authors with citing articles in Table 10 became the most productive authors in the 2010s (Table 11).

### 4.4. Journal Productivity and Citation Impact

The thematic range of journals publishing chemotherapy and oxidative-stress related publications varies over the years. Earlier in the 1990s, journals such as *Biochemical Pharmacology* and *Free Radical Biology and Medicine* were present which mainly publish basic studies or preclinical studies using rodent models. *PLoS One*, a major open access which publishes research from any discipline, shows up in the top journals with citing articles. It was unsurprising to have cancer-focused journals. *Cancer Research* also is a top citing journal which focuses on the broad impact of cancer along with *Anticancer Research* highlighting experimental and clinical oncological studies. *Free Radical Biology and Medicine* persisted into the 2000s as did journals with an oncological focus including the addition of *Oncogene*. *PLoS One* again was the top citing journal in the 2000s, indicating the broad impact of oxidative stress and chemotherapy-related research. The last 10 years have been predominated by open-access journals in both production of publications and citing articles (Table 17 and Table 18). With *Oncotarget* as a top journal, the relevance of oxidative stress as a potential target for cancer is highlighted as well as for other diseases.

### 4.5. Organizational Productivity and Citation Impact

The institutional results coincide with the countries’ data based on the geographical location of the institution. For example, in the 1990s the USA was leading producer and citer of publications which matches with the results of American institutions being both the top producers and citers. The next top regions of Canada and France are represented by the University of Manitoba and Institut National De La Sante et de la Recherche Medicale Inserm (Table 19 and Table 20). The USA institutions continue to dominate the top charts in the 2000s. When China became the most prolific country, we see corresponding Chinese institutions in the top charts. Similarly, with India becoming one of the top producers and citing countries in the 2010s, one of the top institutions was the Council of Scientific Industrial Research in India.

## 5. Conclusions

To conclude, the present bibliometric analysis highlights the research done worldwide concerning oxidative stress as mechanism behind chemotherapy toxicity, especially to the brain. Only recently has chemobrain become a recognized clinical phenomenon affecting the quality of life for not only breast cancer survivors, but also for cancer survivors in general. There is still a need to understand oxidative stress as a mechanism and to research therapeutic antioxidant options.

## Figures and Tables

**Table 1:** Five most productive countries 1990–1999.

Standard Competition Ranking	Country	Articles (%)
First	USA	184 (48.4%)
Second	Japan	33 (8.7%)
Third	Canada	24 (6.3%)
Fourth	France	22 (5.8%)
Fifth	England	20 (5.3%)

**Table 2:** Articles that cite the publications in the analysis 1990–1999. Distribution by country.

No.	Country	Citing Articles 1990–1999	Citing Articles, % of All Countries	Citations Per Publication ^a^
1	USA	6241	37.3	33.9
2	China	1251	7.5	312.8
3	Japan	1153	6.9	34.9
4	Germany	1067	6.4	59.3
5	Italy	892	5.3	44.6
Total of 1–5		10,604	63.3	40.9
All countries		16,747	-	44.1

^a^ Equals citing articles divided by the number of publications from each respective country.

**Table 3:** Five most productive countries 2000–2009.

Standard Competition Ranking	Country	Articles (%)
First	USA	727 (39.1%)
Second	Japan	140 (7.5%)
Third	China	131 (7.0%)
Fourth	India	111 (6.0%)
Fifth	South Korea	98 (5.3%)

**Table 4:** Articles that cite the publications in the analysis 2000–2009. Distribution by country.

No.	Country	Citing Articles 2000–2009	Citing Articles, % of All Countries	Citations Per Publication ^a^
1	USA	23,361	30.0	32.1
2	China	13,411	17.2	102.4
3	Italy	4594	5.9	60.4
4	Japan	4249	5.5	30.4
5	India	4105	5.3	37.0
Total of 1–5		49,720	63.9	26.7
All countries		77,858	-	41.9

^a^ Equals citing articles divided by the number of publications from each respective country.

**Table 5:** Five most productive countries with articles during the period from 2010–2019.

Standard Competition Ranking	Country	Articles (%)
First	China	1930 (29.2%)
Second	USA	1497 (22.6%)
Third	India	659 (10.0%)
Fourth	South Korea	479 (7.2%)
Fifth	Italy	291 (4.4%)

**Table 6:** Articles that cite the publications in the analysis 2010–2019. Distribution by country.

No.	Country	Citing Articles 2010–2019	Citing Articles, % of All Countries	Citations Per Publication ^a^
1	China	1920	29.27	0.07
2	USA	1476	22.50	0.08
3	India	658	10.03	0.13
4	South Korea	474	7.23	0.12
5	Italy	286	4.36	0.06
Total of 1–5		4814	73.4	0.06
All countries		80,625	-	0.08

^a^ Equals citing articles divided by the number of publications from each respective country.

**Table 7:** Five most productive authors 1990–1999.

Standard Competition Ranking	Author	Affiliation	Publications
First	Hasinoff, B.B.	University of Manitoba, Winnipeg, MB, Canada	12
Second	Oberley, L.W.	University of Iowa, Iowa City, Iowa	10
Third	Ferrans, V.J.	National Institutes of Health, Bethesda, Maryland, USA	6
Third	Gutierrez, P.L.	University of Maryland Medical School, Baltimore, Maryland, USA	6
Third	Herman, E.H.	Food and Drug Administration, Laurel, Maryland, USA	6

**Table 8:** Articles that cite the publications in the analysis 1990–1999. Distribution by Author.

No.	Author	Citing Articles 1990–1999	Citing Articles, % of All Authors	Citations Per Publication ^a^
1	Oberley, L.W.	65	0.39	6.5
2	Hasinoff, B.B.	54	0.32	4.5
3	Aggarwal, B.B.	53	0.32	17.7
4	Kang, Y.J.	53	0.32	13.3
5	Li, Y.	52	0.31	NA *
Total of 1–5		277	1.70	9.6
All authors		16,744	-	44.1

^a^ Equals citing articles divided by the number of publications by the same author. * This author did not publish an article that met the search requirements.

**Table 9:** Five most productive authors 2000–2009.

Standard Competition Ranking	Author	Affiliation	Publications
First	Ambrosone, C.B.	Derald H. Ruttenberg Cancer Center, Mount Sinai School of Medicine, New York, New York	18
Second	Oberley, L.W.	University of Iowa, Iowa City, Iowa	17
Third	Spitz, D.R.	University of Iowa, Iowa City, Iowa	16
Fourth	Kalyanaraman, B.	Medical College of Wisconsin, Milwaukee, WI	13
Fifth	Joseph, J.	Medical College of Wisconsin, Milwaukee, WI	12

**Table 10:** Articles that cite the publications in the analysis 2000–2009. Distribution by author.

No.	Author	Citing Articles 2000–2009	Citing Articles, % of All Authors	Citations Per Publication ^a^
1	Zhang, Y.	448	0.58	112.0
2	Wang, Y.	432	0.56	216.0
3	Liu, Y.	414	0.53	207.0
4	Li, Y.	366	0.47	73.2
5	Zhang, L.	332	0.43	66.4
Total of 1–5		1992	2.56	1.1
All authors		77,858	-	41.9

^a^ Equals citing articles divided by the number of publications by the same author.

**Table 11:** Five most productive authors 2010–2019.

Standard Competition Ranking	Author	Affiliation	Publications
First	Zhang, Y.	University of Florida, Gainesville, FL, USA	76
Second	Wang, Y.	Australian Institute for Bioengineering and Nanotechnology, The University of Queensland, Brisbane, Queensland, Australia	64
Third	Liu, Y.	California Pacific Medical Center, Research Institute, San Francisco, CA, USA	62
Fourth	Li, Y.	Shenyang Pharmaceutical University, Shenyang, China	56
Fifth	Li, J.	Center for Applied Chemical Research, Frontier Institute of Science and Technology, Xi’an Jiaotong University, Xi’an, China	54

**Table 12:** Articles that cite the publications in the analysis 2010–2019. Distribution by Author.

No.	Author	Citing Articles 2010–2019	Citing Articles, % of All Authors	Citations Per Publication ^a^
1	Zhang, Y.	914	1.13	12.03
2	Wang, Y.	810	1.01	12.66
3	Liu, Y.	766	0.95	12.35
4	Li, Y.	713	0.88	12.73
5	Wang, J.	670	0.83	17.63
Total of 1–5		3873	4.80	0.59
All authors		80,625	-	12.29

^a^ Equals citing articles divided by the number of publications by the same author.

**Table 13:** Five most productive journals 1990–1999.

Standard Competition Ranking	Journal	Total (%)	IF (1999)
First	*Biochemical Pharmacology*	19 (5%)	2.755
Second	*Cancer Research*	15 (3.9%)	8.614
Third	*Free Radical Biology and Medicine*	12 (3.2%)	4.079
Fourth	*Archives of Biochemistry and Biophysics*	10 (2.6%)	2.386
Fourth	*Chemical Research in Toxicology*	10 (2.6%)	3.47

Retrieved IF data from [43].

**Table 14:** Articles that cite the publications in the analysis 1990–1999. Distribution by journal.

No.	Journal	Citing Articles 1990–1999	Citing Articles, % of All Journals	Citations Per Publication ^a^
1	*Journal of Biological Chemistry*	363	2.2	36.3
2	*Free Radical Biology and Medicine*	353	2.1	29.4
3	*Cancer Research*	263	1.6	17.5
4	*PLoS One*	155	0.9	NA *
5	*Anticancer Research*	153	0.9	17.0
Total of 1–5		1287	7.7	28.0
All Journals		16,744	-	44.1

^a^ Equals citing articles divided by the number of publications from the same journal. * Corresponding journal did not publish any articles matching the search requirements. * NA or not applicable refers to a journal that produces citing articles but does not produce relevant publications that are being cited in the timeframe.

**Table 15:** Five most productive journals 2000–2009.

Standard Competition Ranking	Journal	Total (%)	IF (2009)
First	*Cancer Research*	78 (4.2%)	7.543
Second	*Journal of Biological Chemistry*	64 (3.4%)	5.328
Third	*Free Radical Biology and Medicine*	54 (2.9%)	6.081
Fourth	*International Journal of Cancer*	33 (1.8%)	4.722
Fifth	*Oncogene*	25 (1.3%)	7.135

Retrieved IF data from [44].

**Table 16:** Articles that cite the publications in the analysis 2000–2009. Distribution by journal.

No.	Journal	Citing Articles 2000–2009	Citing Articles, % of All Journals	Citations Per Publication ^a^
1	*PLoS One*	1664	2.14	416.0
2	*Free Radical Biology and Medicine*	984	1.26	18.2
3	*Journal of Biological Chemistry*	860	1.11	13.4
4	*Cancer Research*	644	0.83	8.3
5	*Oncotarget*	582	0.75	NA *
Total of 1–5		4734	6.08	23.7
All Journals		77,858	-	41.9

^a^ Equals citing articles divided by the number of publications from the same journal. * Corresponding journal did not publish any articles matching the search requirements. * NA or not applicable refers to a journal that produces citing articles but does not produce relevant publications that are being cited in the timeframe.

**Table 17:** Five most productive journals 2010–2019.

Standard Competition Ranking	Journal	Total (%)	IF (2018/2019)
First	*PLoS One*	198 (3.0%)	2.9
Second	*Oncotarget*	148 (2.2%)	3.7
Third	*Scientific Reports*	117 (1.8%)	4.1
Fourth	*Free Radical Biology and Medicine*	107 (1.6%)	5.5
Fifth	*International Journal of Molecular Sciences*	85 (1.3%)	4.2

Retrieved IF data from [45].

**Table 18:** Articles that cite the publications in the analysis 2010–2019. Distribution by journal.

No.	Journal	Citing Articles 2010–2019	Citing Articles, % of All Journals	Citations Per Publication ^a^
1	*PLoS One*	1563	1.94	7.89
2	*Oncotarget*	1412	1.75	9.54
3	*Scientific Reports*	1360	1.69	11.62
4	*International Journal of Molecular Sciences*	1181	1.47	13.89
5	*Molecules*	714	0.89	19.30
Total of 1–5		6230	7.73	0.95
All Journals		80,625	-	12.29

^a^ Equals citing articles divided by the number of publications from the same journal.

**Table 19:** Five most productive organizations 1990–1999.

Standard Competition Ranking	Organizations	Articles (%)
First	National Institutes of Health (NIH), USA	24 (6.3%)
Second	University of Texas System	16 (4.2%)
Third	NIH National Cancer Institute (NCI)	14 (3.7%)
Third	University of Manitoba, Canada	14 (3.7%)
Fourth	University of Minnesota System	12 (3.2%)

**Table 20:** Articles that cite the publications in the analysis 1990–1999. Distribution by institutions.

No.	Institution	Citing Articles 1990–1999	Citing Articles, % of All Institutions	Citations Per Publication ^a^
1	University of Texas System	461	2.8	28.8
2	National Institutes of Health (NIH)	422	2.5	17.6
3	University of California System	414	2.5	51.8
4	Institut National De La Sante et de la Recherche Medicale Inserm	277	1.7	55.4
5	Harvard University	249	1.5	62.3
Total of 1–5		1823	10.9	32.0
All Institutions		16,744	-	44.1

^a^ Equals citing articles divided by the number of publications from the same institution.

**Table 21:** Five most productive organizations 2000–2009.

Standard Competition Ranking	Organizations	Articles (%)
First	National Institutes of Health (NIH), USA	73 (3.9%)
Second	NIH National Cancer Institute (NCI)	51 (2.7%)
Third	University of Texas System	49 (2.6%)
Fourth	University of California System	42 (2.3%)
Fifth	University of Iowa	40 (2.2%)

**Table 22:** Articles that cite the publications in the analysis 2000–2009. Distribution by institution.

No.	Institution	Citing Articles 2000–2009	Citing Articles, % of All Institutions	Citations Per Publication ^a^
1	University of Texas System	2020	2.59	41.22
2	University of California System	1693	2.17	40.31
3	National Institutes of Health (NIH), USA	1491	1.92	20.42
4	Harvard University	1339	1.72	44.63
5	Institut National De La Sante et de la Recherche Medicale Inserm	1204	1.55	44.59
Total of 1–5		7747	9.95	4.17
All Institutions		77,858	-	41.88

^a^ Equals citing articles divided by the number of publications from the same institution.

**Table 23:** Five most productive organizations 2010–2019.

Standard Competition Ranking	Organizations	Articles (%)
First	Chinese Academy of Sciences	159 (2.4%)
Second	University of Texas System	142 (2.1%)
Third	Council of Scientific Industrial Research CSIR India	124 (1.9%)
Fourth	Shanghai Jiao Tong University	86 (1.3%)
Fifth	UTMD Anderson Cancer Center	85 (1.3%)

**Table 24:** Articles that cite the publications in the analysis 2010–2019. Distribution by institution.

No.	Institution	Citing Articles 2010–2019	Citing Articles, % of All Institutions	Citations Per Publication ^a^
1	Chinese Academy of Sciences	2332	2.89	14.76
2	University of Texas System	1449	1.80	10.50
3	Harvard University	1270	1.58	17.40
4	University of California System	1235	1.53	17.64
5	Shanghai Jiao Tong University	1058	1.31	12.30
Total of 1–5		7344	9.11	1.12
All Institutions		80,625	-	12.29

^a^ Equals citing articles divided by the number of publications from the same institution.

## Supplementary Materials

The following are available online at https://www.mdpi.com/2076-3921/9/5/439/s1, Table S1: Thesaurus file; Table S2: Term map data 1990–1999; Table S3: Term map data 2000–2009; Table S4: Term map data 2010–2019; and Table S5: World map data.

## Abbreviations

VEGF	Vascular endothelial growth factor
ROS	Reactive oxygen species
CNS	Central nervous system
SOD	Superoxide dismutase
NIH	National Institutes of Health
NCI	National Cancer Institute
CSIR	Council of Scientific Industrial Research
UTMD	University of Texas MD
Nrf2	Nuclear factor erythroid-derived 2 related factor
Keap1	Kelch-like ECH-associated protein 1
DOX	Doxorubicin
NAC	N-acetylcysteine
NP	Nanoparticles
ts	Topic search
